# Sexual Identity, Gender-Nonconformity, and Acoustic Speech Characteristics of Gay and Straight Australian English-Speaking Men

**DOI:** 10.1007/s10508-026-03468-4

**Published:** 2026-06-27

**Authors:** Tünde Szalay, Duy Duong Nguyen, Paolo Bravi, Antonia M. Chacon, John Holik, Grace Leonard, James S. Morandini, Catherine J. Madill

**Affiliations:** 1 Sydney Voice Lab, Discipline of Speech Pathology, Faculty of Medicine and Health, Sydney School of Health Sciences, The University of Sydney, D18 Susan Wakil Health Building, Western Avenue, Camperdown, Sydney, NSW 2050 Australia; 2 Department of Humanities and Social Sciences, University of Sassari, Nuremberg, Germany; 3 School of Psychology, The University of Sydney, Sydney, Australia; 4 King Street Psychology Clinic, Sydney, Australia

**Keywords:** LGBTQI+, Masculinity, Fundamental frequency, Fricatives, Vowels, Sexual orientation

## Abstract

Gender-nonconforming acoustic speech features, such as fundamental frequency, spectral moments of the fricative /s/, and vowel formants produced at higher frequencies, relative to straight men, have been examined as markers of gay identities in multiple varieties of English. Production of these features is not observed consistently, as some gay men exhibit some gender-nonconforming features without producing globally gender-nonconforming speech. Variation in speech features is further increased by accent differences and linguistic diversity in the gay community. This study investigated acoustic characteristics of gay speech in Australian English (AusE), a variety with limited experimental research on gay speech. Acoustic characteristics of fundamental frequency, /s/, and vowel production were compared between 35 gay and 25 straight AusE-speaking men. Speech variation among gay men was examined in relation to gender-nonconformity, masculinity, self-identified straight-acting behavior, outness, and internalized homophobia. AusE-speaking gay men produced fundamental frequency with gender-nonconforming larger variation and /s/ with gender-nonconforming higher center of gravity than straight men. Vowel production with gender-nonconforming, higher formants indexed both gay identity and gay men who identified as less masculine and less straight-acting.

**Supplementary Information:**

The online version contains supplementary material available at 10.1007/s10508-026-03468-4.

## Introduction

In several languages, including Australian English (AusE), speech research has found that some gay men^1^ differ from straight men on speech features that are stereotypically or empirically associated with women’s speech or with gender-nonconforming male speech (Linville, 1998; Munson et al., 2006; Pierrehumbert et al., 2004; Shea et al., 2024; Sulpizio et al., 2015; Szalay et al., 2023; cf. with Geng & Gu, 2022; Holmes et al., 2024). Importantly, these differences are often feature-specific rather than global, with gay men showing gender-nonconforming patterns on some speech variables but not others (Munson et al., 2006; Pierrehumbert et al., 2004). Prior researchers have sometimes interpreted this pattern in terms of “selective adoption” of particular speech features; however, the causal mechanisms underlying such patterns remain unresolved (Daniele et al., 2020; Munson et al., 2006; Pierrehumbert et al., 2004; Podesva, 2007).

Multiple gender-nonconforming acoustic features have been compared and identified as markers of gay-straight differences in several languages, such as gender-nonconforming /s/ production (as in *see, saw),* while other acoustic features, such as vocal fundamental frequency (perceived as pitch), are less likely to show gay-straight differences (Linville, 1998; Sulpizio et al., 2015; for an overview, see Simpson & Weirich, 2020). In AusE, however, only two studies examined acoustic features of gay speech with respect to /s/ and creaky voice production (Shea et al., 2024; Szalay et al., 2023). Therefore, this study examined a range of acoustic features to determine which gender-nonconforming speech features are exhibited by AusE-speaking gay men and how speech features are linked to gender-nonconformity.

The majority of studies compared gay and straight speakers at a group-level (Linville, 1998; Munson et al., 2006; Pierrehumbert et al., 2004; Rendall et al., 2008; Sulpizio et al., 2015). As group-level comparison was criticized for erasing linguistic diversity within the gay community (Podesva, 2007), now an increasing body of research has examined how gay speech differs within the community, and what speaker-characteristics (e.g., gender-nonconformity, political views, urban/rural orientation) drive differences between gay individuals (Kachel et al., 2018; Levon, 2009; Podesva & Hofwegen, 2014; Shea et al., 2024).

Gender-nonconformity may be important for understanding speech differences both between gay and straight men and among gay men themselves. More broadly, gay men have been found on average to be more gender-nonconforming than straight men across a range of measures, and this broader variation may also be reflected in speech (Bailey & Zucker, 1995; Kachel et al., 2018; Lippa, 2020; Podesva & Hofwegen, 2014; Rieger et al., 2008; Shea et al., 2024). For example, some gay speakers of Generalized American English have been found to diverge from gender-conforming masculine speech by producing higher-frequency /s/ sounds (Linville, 1998). However, rural gay speakers of North California English did not show this pattern and instead patterned with straight men on /s/ production (Podesva & Hofwegen, 2014). Podesva and Hofwegen suggested that this difference may reflect broader differences in gender conformity across communities, with the urban university sample drawn from a context with more relaxed gender norms than the rural sample. Taken together, these findings suggest that speech differences among gay men may be better understood in the context of broader variation in gender-nonconformity, rather than assumed to be uniform across speakers or communities.

### Sex and Gender Differences Between Men’s and Women’s Speech

Some speech differences between men and women, which were examined as indices of gay identity in men, are grounded in sexual dimorphism (Munson & Babel, 2019). However, due to speakers’ ability to control their articulators, differences can be intentionally exaggerated, such that speech differences are larger than what is expected based on anatomical differences, weakening the correlation between gendered speech and sexual dimorphism (Johnson, 2006; Munson and Babel, 2019). Other speech differences are arbitrary, indicating that they are learned (Munson & Babel, 2019).

The higher F0 (the acoustic measure of the vibration of the vocal folds) of women is grounded in women having smaller and lighter vocal folds that vibrate faster, increasing F0 (Leung et al., 2022; Munson & Babel, 2019). Yet, F0 differences between men and women are more pronounced in Japanese than in Dutch (van Bezooijen, 1995; Yamazawa & Hollien, 2009), consistent with sex differences being exaggerated due to Japanese culture placing higher value and more positive perception of an F0 that matches one’s biological sex relative to Dutch (van Bezooijen, 1995).

The higher center of gravity of /s/ (i.e., the first spectral moment capturing mean concentration of acoustic energy) in women’s speech is grounded in women having smaller anterior oral cavities during /s/ production than men (Jongman et al., 2000; Stevens, 2000; Stevens & Harrington, 2016). The variance and kurtosis (the second and fourth spectral moments, capturing the range and peakedness of distribution of acoustic energy respectively) are also higher in women’s than in men’s speech, while skewness (the third spectral moment, capturing the asymmetry of the distribution) is lower (Jongman et al., 2000). However, differences based in sexual dimorphism are attenuated in Glasgow English, where working class men and women show reduced /s/ differences compared to middle class men and women, consistent with differences in /s/ production being controlled by speakers (Stuart-Smith, 2007). Similarly, prepubescent children as young as 3 years old show gender differences in /s/ production, earlier than sexual dimorphism develops in puberty, consistent with gendered speech differences being learned (Wong et al., 2025).

Women’s higher vowel formants (i.e., concentration of acoustic energies in a specific frequency range carrying information on speaker characteristics and vowel identity, e.g., contrasting /iː/ in *heed* vs. /e/ in *head*) are grounded in women having approximately equal oral and pharyngeal cavities while men have larger pharyngeal than oral cavities (Fant, 1966; Fitch & Giedd, 1999). However, the degree of gender differences varies by language, suggesting that differences grounded in sexual dimorphism can be exaggerated and attenuated as speakers control their articulators (Johnson, 2006; Munson & Babel, 2019). For example, speakers can increase the size of their anterior oral cavities by rounding their lips and thus amplify lower frequency ranges (Munson & Babel, 2019).

### Speech Characteristics of Gay Men Relative to Straight Men

Acoustic characteristics of gay speech have been examined in multiple varieties of English as well as in other languages to determine which gender-nonconforming features are produced: higher mean F0, wider F0 range; the fricative /s/ produced with higher center of gravity and longer duration; and higher vowel formants (Table 1). Higher F0 and wider F0 range were one of the first acoustic characteristics examined with respect to gay sexual identity due to the stereotype of gay speakers producing these gender-nonconforming voice patterns (Gaudio, 1994). However, acoustic-phonetic studies failed to find a link between higher F0 and wider F0 range and self-reported sexual identity in a series of studies on American and Canadian English, German, and Czech, providing no evidence for gay men producing higher and more varied F0 (Gaudio, 1994; Munson et al., 2006; Rendall et al., 2008; Sulpizio et al., 2015; Valentova & Havlíček, 2013). Gay speakers of British English and Mandarin Chinese produced lower F0 than straight speakers, attributed to the speakers’ desire to be viewed positively or to mask their gay identities respectively (Geng & Gu, 2022; Holmes et al., 2024). In Dutch, gay speakers produced higher F0, while gay speakers of French produced more varied F0 without an overall F0 increase (Baeck et al., 2011; Suire et al., 2020). These studies together highlight diversity and variation in markers of what was once thought of as “gay speech.”

**Table 1:** Overview of the evidence for acoustic differences between gay and straight male speakers of English

Study	Accent	Mean F0	F0 range	Voice modality	/s/ center of gravity	Vowel formants
Predicted by selective feature adoption		Higher	Wider	No clear prediction	Higher	Higher
Linville (1998)	American English	No difference			Higher	
Munson et al. (2006)	American English		No difference		No difference	Higher formants in /æ/ and /ɛ/
Pierrehumbert et al. (2004)	American English					F1 in /ɑ/, F2 in /i:/, and F1 and F2 in /æ/ were higher; F2 in /ɑ/, F1 in /i/ were lower
Rendall et al. (2008)	Canadian English	No difference				Higher formants in /ʌ/ and /uː/
Szalay et al. (2023)	Australian English				Higher	
Shea et al. (2024	Australian English			Higher creak prevalence		
Holmes et al. (2024)	British English	Lower	Narrower			No difference

Empty cells indicate that the acoustic feature was not examined by the study

The production of /s/ with an increased frequency range and longer duration are prominent stereotypes of a gender-nonconforming speech feature associated with gay speakers (Sulpizio et al., 2015; Van Borsel & Van de Putte, 2014). However, there is no consistent evidence that gay speakers produce /s/ with higher center of gravity, lower skewness, and longer duration. A smaller cohort of American English gay speakers produced /s/ with higher center of gravity and longer duration, whereas a larger cohort produced /s/ with lower skewness (Linville, 1998; Munson, 2007; Munson et al., 2006). Preliminary results on /s/ production by Australian English speakers indicate that gay speakers produce /s/ with a higher center of gravity (Szalay et al., 2023).

In vowel production, gender-nonconforming patterns by gay speakers would be evident through higher F1 and F2 values relative to straight men (Munson et al., 2006; Pierrehumbert et al., 2004; Rendall et al., 2008). Higher formant values, however, were only observed for the first formant of some vowels in American and Canadian English (American English: /æ, ɛ/; Canadian English: /ʌ, uː/), but not consistently across all vowels (Munson et al., 2006; Rendall et al., 2008).

These studies collectively suggest that some gay speakers produce some, but not all speech features in a gender-nonconforming manner, often with inconsistent results, as gay speakers produce different gender-nonconforming features in different languages, accents, and communities (Table 1). The complexity of “gay speech” is consistent with selective adoption of gender-nonconforming speech features (Munson et al., 2006; Pierrehumbert et al., 2004). However, treating gay and straight as binary categories when searching for features that index gay speech is potentially problematic (Levon, 2021). For example, when sexual identity was measured using the Kinsey-scale instead of binary categories, German speakers identifying as exclusively straight differed in /s/ production from speakers identifying as mostly straight, while exclusively gay speakers differed from mostly gay speakers in terms of jitter (Kachel et al., 2018).

### Speech Features, Gender-Nonconformity, and Other Aspects of Gay Identities

Phonetic features of gay speech may index multiple aspects of the speaker’s psychological characteristics, such as gender-nonconformity, masculinity, and outness (Kachel et al., 2018; Levon, 2021; Munson, 2007; Shea et al., 2024). Self-perceived masculinity has been correlated with gender-conforming speech patterns produced by German gay speakers (Kachel et al., 2018). Gay speakers who described themselves as more masculine, produced more masculine lower center of gravity in /s/ and lower first and second formant in /uː/ (Kachel et al., 2018). Gay speakers who described themselves as more feminine produced the more gender-nonconforming higher center of gravity in /s/ and higher first and second formant in /uː/ (Kachel et al., 2018).

In AusE, the links between gay sexual identity, attitudes to traditional masculinity, and speech were examined through creaky voice, i.e., voice produced with low and irregular F0, also referred to as vocal fry (Shea et al., 2024). Gay speakers of AusE, consistent with the stereotypes of creak being feminine, produced more creak than straight men (Shea et al., 2024). In contrast with the speech stereotypes, gay men holding more traditional views on masculinity produced *more* creak than gay men who do not hold traditional views (Shea et al., 2024). The authors proposed that gay men who agreed with traditional masculinity used more creak to lower their F0, and thus present themselves as more masculine (Shea et al., 2024). These results suggest that attitudes to masculinity can partly account for speech variation in the AusE-speaking gay community (Shea et al., 2024).

The connection between speech and how open gay men are about their gay identity, colloquially referred to as “outness” is less well understood (Daniele et al., 2020; Kachel et al., 2018). A perceptual study conducted using samples of English-speaking YouTubers showed that gay YouTubers are more likely to be perceived as gay in videos they published after coming out publicly compared to videos they published prior to coming out (Daniele et al., 2020). An acoustic study on German gay speakers did not find an effect of outness on gender-nonconforming speech patterns (Kachel et al., 2018).

These results combined indicate that in addition to examining differences between gay and straight speech, other relevant sexuality and identity constructs need to be considered when examining acoustic features indexing gay identity. For example, contact with female speakers in childhood in German speakers, or more progressive political views in Hebrew were found to increase the likelihood of gender-nonconforming speech production by gay speakers (Kachel et al., 2018; Levon, 2009). The effect of other factors, such as self-reported straight-acting behavior (gay males who identify as stereotypically masculine in presentation) or internalised homophobia, has not been examined, although these factors also may affect speech through their association with gender expression (Hunt et al., 2020).

In AusE, to the best of our knowledge, only two studies have addressed acoustic differences between gay and straight speakers, examining differences in /s/ production (Szalay et al., 2023) and creaky voice (Shea et al., 2024), with the former comparing gay men to straight men, and the latter considering sexual identity as well as attitudes to masculinity. Yet, the diversity of acoustic markers indexing gay identities in other accents of English demonstrates that gay speech cannot be characterised by /s/ and voice production alone, necessitating an overview of multiple acoustic markers.

### The Present Study

We aimed to extend our understanding of acoustic features indexing gay identity in Australian English by providing a sociophonetic overview of gender-nonconforming F0 estimates, fricative spectral moments, and vowel formant estimates. Gender-nonconforming speech in men was defined as a shift toward patterns more typical in women’s speech on each acoustic dimension (Table 1). Specifically, gender-nonconforming speech in men was operationalised as less male typical F0 (perceived as pitch) with higher mean and greater variability (larger SD and range), /s/ spectral moments (higher center of gravity, higher spectral SD, lower skewness, higher kurtosis, and longer duration), and vowel formants (higher F1 and higher F2). In addition, we aimed to explore important variation within gay speech based on certain sexual identity variables.

Firstly, acoustic differences were examined at a group level between male speakers identifying as gay and straight. Secondly, acoustic differences within gay speakers were linked to several psychological speaker characteristics, such as mostly versus exclusively gay identity, self-perceived gender-nonconformity, and factors related to attitudes toward and social integration of one’s gay identity, i.e., outness, self-reported straight-acting behavior, and internalised homophobia. We hypothesised that

1. Gay men would produce more gender-nonconforming speech features relative to straight men, in terms of:
  - higher mean F0, larger F0 SD, and larger F0 range,
  - /s/ with higher center of gravity, higher spectral SD, lower skewness, higher kurtosis, and longer duration
  - vowels with higher F1 and higher F2.
2. There would be within group variability among gay men such that
  - Gay men who rate their sexual identity as “exclusively gay” would produce more gender-nonconforming acoustic markers than those rating their sexual identity as less exclusively gay (i.e., “mostly gay”).
  - Gay men who perceive themselves as less masculine would produce more gender-nonconforming acoustic markers
  - Sexual identity variables related to greater integration of a gay identity (i.e., greater outness, lower internalized homophobia) would be related to producing more gender-nonconforming acoustic markers.

## Method

The current study analyzed a subset of audio samples collected for a larger project between 2015 and 2016 to examine sexual identity, gender expression, and biomarkers in men. For details of the original study, see Hunt et al. (2020); for details of inclusion criteria in the current study, see the section Speech Data Selection and Screening.

### Participants

To be eligible for the original project, participants had to be male, 18 years or older, and residents of Australia. Gay participants were recruited via pop-up advertisements on a large social media application targeted at men who have sex with men. Straight men (n = 300) were recruited via Qualtrics panels. Participation of both groups were incentivized with monetary prizes and reimbursement. In total, 2133 participants participated in the study (gay = 1546, bisexual = 287, straight = 300). Amongst these, 229 gay and bisexual participants and 243 straight participants successfully uploaded a voice sample.

Only participants who identified categorically as “Gay” or “Straight” and who subsequently (on a separate, Kinsey-like, five-point continuum) rated their sexual identity as “Gay” or “Mostly gay” or “Straight” or “Mostly straight” were considered (see section Survey Data Collection and Analysis).

After audio data screening (see section Speech Data Selection, Quality, and Validity), speech data from 60 participants (gay = 35 participants, mean age with standard deviation = 33.6 (11.6); straight = 25 participants, mean age with standard deviation = 36.2 (14.5)) were included for analysis in the present study.

### Procedure

#### Speech Material and Recording

For voice recordings, participants were asked to recite the first four lines of the lyrics of “Advance Australia Fair,” the national anthem of Australia: “Australians all let us rejoice / For we are young and free/We’ve golden soil and wealth for toil / Our home is girt by sea” (Australian National Anthem | PM&C, 2022). This speech task was chosen as participants were not expected to be able to simultaneously read from their smartphone screen and use a recording application but were expected to be able to recite the first four lines of the national anthem of Australia.

Participants were instructed to recite the text using their most comfortable speaking voice without singing. Participants were instructed to record in a quiet location where possible. Audio samples were recorded either as audio files (mp3) or audio-visual files (mp4) using participants’ smartphones and uploaded using the file-upload function in Qualtrics. Included voice samples were approximately 10 s in length and had a sampling rate of 44,100 Hz or 48,000 Hz.

### Acoustic Phonetic Analysis

#### Speech Data Selection, Quality, and Validity

Voice samples were recorded at uncontrolled locations via smartphones, known to pose challenges for speech data collection and analysis due to reduced audio quality and increased variation between participants (De Decker & Nycz, 2011; Leemann et al., 2020; Penney et al., 2021; Rathcke et al., 2017). Location and smartphone recordings affect audio quality as background noise and lossy compression in mp3 and mp4 recordings reduce quality relative to lab-based recordings, while the differences between each participant’s recording location and device add uncontrolled variation to the data (Bulgin et al., 2010; De Decker & Nycz, 2011; Leemann et al., 2020). As recording device and location were not controlled in the current study, a number of steps were taken to ensure that the audio signals used were suitable for acoustic-phonetic analysis and the extracted measures were valid.

Firstly, voice clinicians (Authors DDN, AC, and CM, two of whom are native listeners of AusE) screened and signal-typed the uploaded voice samples in an iterative consensus procedure to ensure that (1) participants recited the Australian anthem without singing; (2) participants recited at least three out of the expected four lines of the anthem; (3) participants spoke AusE; and (4) speech was suitable for acoustic analysis (e.g., appropriate volume, no audible background noise, no signal distortion, clipping, or muffling). Screening and signal-typing were conducted using auditory-impressionistic methods and visual observations of all spectrograms; audio files not meeting these criteria were excluded from further analysis.

Secondly, all included audio samples were high-pass filtered at 500 Hz and a 6-dB roll-off using Audacity to reduce the effect of background noise prior to estimating acoustic characteristics (Audacity Team, 2021). The filter did not eliminate sound below the 500 Hz cut-off frequency; instead, it attenuated the signal by 3 dB at 500 Hz and then by 6 dB at 250 Hz, with minor effects on F1. Signal attenuation continued linearly at 6 dB/octave, such that signal magnitude at 125 Hz and 62.5 Hz was attenuated by 12 dB and 18 dB respectively, affecting F0. The sound was increasingly attenuated by 18 dB and more below 62.5 Hz, in which range no acoustic markers were expected. After high-pass filtering, the signal to noise ratio of the included sound files was 25 dB on average (straight speakers: mean = 23 dB, SD = 3 dB; gay speakers: mean = 26 dB, SD = 4 dB). This signal to noise ratio is considered typical for mobile phone data, allowing for acoustic analysis (Guan & Li, 2021).

Lastly, acoustic measures estimated in the recordings were compared to reference values recorded under laboratory circumstances produced in citation forms. This was to ensure validity of the acoustic measures estimated in smartphone recordings taken at uncontrolled locations. AusE is a well-studied variety of English, with reference values available on the acoustic markers examined here: F0 (Leung et al., 2022), /s/ (Stevens & Harrington, 2016) F1 and F2 (Cox, 1999, 2006; Cox & Penney, 2024).

#### Fundamental Frequency Estimation and Validation

Fundamental frequency (F0) data were estimated from the entire utterance, (n = 60 samples from 60 participants in total), starting from the first word of the anthem and ending with the last word produced by the participant (last word of the third or fourth line). The start and end of the first and the last word were determined based on a sudden increase in amplitude.

F0 contour was estimated using the *To Pitch (filtered ac)* function in Praat that applied a low-pass filter with a 3% attenuation at formant ceiling (Boersma & Weenink, 2023). The F0 ceiling was set to 300 Hz and floor to 75 Hz, as the normative values for AusE men were reported as a minimum of 80 Hz and a maximum of 258 Hz (Leung et al., 2022). The remaining F0 settings were set to default, such that F0 contour was estimated throughout the vowel at every 0.001 s with 15 candidates, a silence threshold of 0.09, voicing threshold of 0.5, voiced/unvoiced cost of 0.14, octave cost of 0.055 per octave and octave jump cost of 0.35. F0 contours overlaid on spectrograms were observed visually. F0 measures produced during creaky periods were included. Three F0 characteristics were estimated in the F0 contour: mean and standard deviation of F0, as well as range of F0, calculated as the difference of maximum and minimum F0, yielding a total of 3 (characteristics) × 60 (utterance) = 180 F0 estimates.

F0 values were validated by comparing F0 measurements of straight speakers estimated in the current dataset to reference values of AusE extracted from a read passage using one-sample *t*-tests (Table 2, Leung et al., 2022). Results show that mean, standard deviation, and range F0 was significantly lower (*p* =.035, *p* <.0001, and *p* <.0001 respectively) in the current dataset relative to reference values. The 6 Hz difference in mean F0 between reference values and straight speakers in the current dataset was not considered phonetically meaningful (Table 2.). The significant differences in standard deviation and range are attributed to potential outlier value(s) in the reference data (Leung et al., 2022). In the reference data, a high standard deviation of 31 Hz and a wide range of 169 Hz is reported for the age range 30–39, while other age-ranges report a standard deviation of 16–18 Hz and a range of 59–72 Hz, comparable to the current dataset.

**Table 2:** Descriptive statistics of F0 characteristics of gay and straight male speakers compared against normative values of AusE-speaking male speakers

	Leung et al. (2022)	Straight speakers	Gay speakers
Mean (Hz)	115	109	107
Standard deviation (Hz)	21	11	14
Range (Hz)	178	75	77

#### Fricative Measurement and Validation

The fricative /s/ was identified in two words (*us, rejoice*) per utterance giving a total of 2 (words) × 60 (utterances)–6 (excluded words) = 114 (Table 3). Words were excluded due to the target words containing the fricatives being misquoted. Fricative boundaries were located automatically using the MAUS forced aligner with the AusE grapheme-to-phoneme converter (Kisler et al., 2017; Schiel, 1999, 2015) and corrected manually in a *Praat* interface based on the onset and offset of high-intensity noise (Boersma & Weenink, 2023). The 50–75% (i.e., the third quarter) of the fricative was identified to estimate spectral moments, as center of gravity reaches its peak in the third quarter (Jongman et al., 2000; Stevens & Harrington, 2016). The third quartile was extracted and converted into a frequency spectrum with fast Fourier transformation using Praat (Boersma & Weenink, 2023). Center of gravity, standard deviation, skewness, and kurtosis were estimated.

**Table 3:** Number of extracted phonemes and their carrier words

Phoneme	Word(s)	No. of tokens	Acoustic metrics
/iː/	*we, free, sea*	171	
/e/	*let*	56	
/oː/	*all, for* (conjunction)	113	F1, F2
/ɐ/	*young*	60	
/ɐː/	*are*	59	
/s/	*us, rejoice*	120	Center of gravity, standard deviation, skewness, kurtosis

The words *for* and *are* are pronounced without an /ɹ/ as AusE is a non-rhotic accent

Center of gravity values were compared to reference values of /s/ at 5500 Hz for AusE-speaking men (Stevens & Harrington, 2016). Mean center of gravity was 5323 Hz (standard deviation = 1625 Hz) for straight and 6168 Hz (standard deviation = 1971 Hz) for gay speakers in the current data. A one-sample *t*-test showed that the 177 Hz difference between straight speakers in the current data and reference values was not statistically significant (*p*-value =.4658; Stevens & Harrington, 2016).

#### Formant Estimation and Validation

For each of the 60 audio samples, the vowels /iː, e, oː ɐ, ɐː/ (*we, sea, free; let; all, for*- conjunction; *young; are*) were identified to outline the AusE vowel space.^2^ The selected vowels were extracted from eight words; words were excluded when they were misquoted (Table 3). High lexical frequency words carrying word-level and sentence-level stress were preferred when available to reduce the likelihood of misquoting and vowel reduction.

F1 and F2 values were estimated using a semi-automatic method. Vowel boundaries were located automatically using the MAUS forced aligner with the AusE grapheme-to-phoneme converter (Kisler et al., 2017; Schiel, 1999, 2015) and corrected manually in a Praat interface based on sudden changes in amplitude, periodicity, voicing, and visual inspection of formant trajectories (Boersma & Weenink, 2023).

**Fig. 1:**
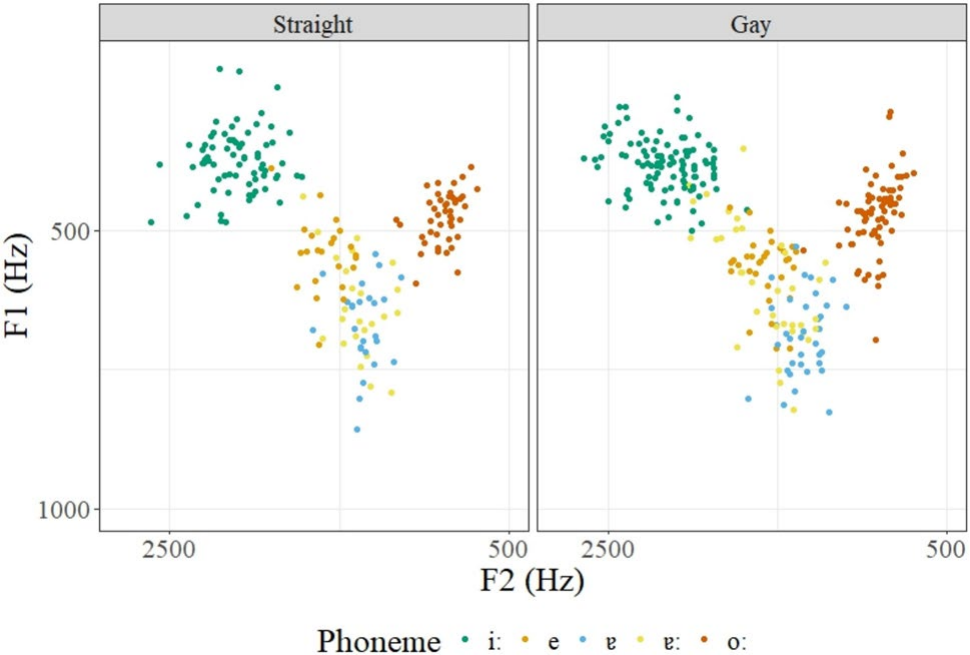
Vowel space of gay and straight male speakers of AusE. Colours indicate vowel phonemes

Formant estimation was carried out at a single timepoint, at the vowel target identified by visual inspections of the formants’ inflection points. When formant changes were not observed, vowel target was placed at the acoustic midpoint of the vowel. Formant measurements at vowel target were extracted automatically and corrected manually in Praat (Boersma & Weenink, 2023). Formant frequencies were estimated using the *To Formant* function of Praat at every 5 ms throughout a 25 ms formant analysis window using a 50 ms Gaussian window with 75% overlap and a pre-emphasis filter increasing spectral slope above 100 Hz by 6 dB/octave. For each speaker, 5 formants were tracked up to 5000 Hz ceiling, and for 5500 Hz for one participant producing higher formant values. Formant measurements were manually corrected using a Praat-based interface that superimposed formant estimates over a broadband spectrogram calculated over 5 ms windows with 40% overlap, allowing for corrections of estimates that did not align with the visible formants (Szalay et al., 2022). Visualisation of vowel formants showed that the F1-F2 measures reflected the expected shape of the AusE vowel space for both straight and gay speakers (Fig. 1).

### Survey Data Collection and Validation

Participants responded to demographic questions (e.g., age, ethnic background, religiosity), and survey items about their gender expression, attitudes to their sexual identity, and several other scales not relevant to the current study. Survey items were presented in a fixed order.

Survey items related to speakers’ attitudes towards their sexual identity and masculinity were included in the current analysis (for detailed analysis of the survey data, see Hunt et al., 2020). The survey item assessing sexual identity as a categorical variable was selected to examine group-level acoustic differences between straight and gay speakers. To examine acoustic variation among the gay speakers, the survey item assessing sexual identity as a continuum, and items assessing gender expression and attitudes to own sexual identity were selected. Survey data analysis was conducted in SPSS.

#### Items Assessing Sexual Identity

Two items assessed elements of sexual identity. The first, a categorical measure of sexual identity label, asked participants “What sexual orientation do you most identify with at present?” Responses included “gay”, “bisexual”, “queer”, “pansexual”, “questioning”, “straight”, or “other” (with participants asked to clarify). Only participants reporting “gay” or “straight” sexual identities were included in the current study. The second, a sexual identity continuum, asked participants to rate their sexual identity on a five-point scale from “gay”, “mostly gay”, “bisexual”, “mostly straight”, to “straight”. Only participants reporting “gay/mostly gay” or “straight/mostly straight” sexual identities were included in the current study. Sexual Identity responses as “straight” or “gay” were coded as binary 0 and 1 respectively, while the Five Point Sexual Identity Continuum was coded from Straight as 1 to Gay as 5.

#### Items Assessing Gender Expression

Straight and gay participants’ self-reported masculinity-femininity was assessed using two identical items—the first assessing self-reported Masculinity-Femininity in Childhood and the second assessing Masculinity-Femininity in Adulthood. Responses were rated on a 7-point Likert scale ranging from 1 (very feminine) to 7 (very masculine) in response to the questions “How feminine or masculine do you feel as a man?” and “How feminine or masculine did you feel as a boy?” (Sánchez et al., 2010; Smolenski et al., 2010).

Childhood Gender-Nonconformity was assessed on three items from the Childhood Gender-Nonconformity scale (Bailey & Oberschneider, 1997). Items required participants to indicate their endorsement with gender-atypical interests and behaviors in childhood such as “I was a feminine boy.” Endorsement of each item was indicated using a 7-point scale ranging from strongly disagree to strongly agree. High scores were associated with higher levels of gender-nonconformity. The three items measuring childhood gender-nonconformity demonstrated good internal consistency among gay (α = 0.72), as well as among straight men (α = 0.71), allowing for using the mean rating across the three items for each participant.

Straight-acting Identification assessed participants’ perception of themselves as identifying with traditional, heteronormative masculinity, i.e., “straight-acting” (Hunt et al., 2020). Straight-acting identification was evaluated using a single item “I see myself as straight-acting” with responses registered on a 5-point Likert scale, ranging from 1 (strongly disagree) to 5 (strongly agree).

#### Items Related to Gay Participants’ Sexual Identity

Outness assessed the level of disclosure of one’s sexual orientation and was assessed across multiple relationship types, using three items from Hunt et al. (2012). Specifically, participants rated three items on how open they were about being gay with (1) their family, (2) their friends, and (3) their co-workers and employers on a 5-point scale (1 = Completely hidden, 5 = Completely open). The three items measuring outness demonstrated good internal consistency for men identifying as gay (α = 0.860), allowing for using the mean rating across the three items for each participant.

Internalized Homophobia was assessed using the 7-item Reactions to Homosexuality Scale-Short Form (Smolenski et al., 2010). The scale assesses three factors relevant to how gay men feel about their sexual identity, namely Personal Comfort with a Gay Identity (e.g., “Even if I could change my sexual orientation, I wouldn’t”), Social Comfort with Gay Men (e.g., “Social situations with gay men make me feel uncomfortable”), and Public Identification as Gay (e.g., “I feel comfortable being seen in public with an obviously gay person”). All items were rated using a 7-point Likert scale, ranging from 1 (strongly disagree) to 7 (strongly agree). Items of the Internalized Homophobia scale demonstrated good internal consistency (α = 0.78), allowing for using the mean rating across the three items for each participant.

### Statistical Analysis

Statistical analysis was carried out in R (R Core Team, 2021). Demographic differences were tested using one-sided *t*-tests. To test the hypotheses that gay men would produce gender-nonconforming speech features in acoustic characteristics of (1) F0 estimates, (2) /s/ spectral moments and duration, and (3) vowel formant estimates, three logistic regression models were built using Sexual Identity as response variable (binomial, straight coded as 0 and gay coded as 1) and acoustic measurements as the independent variables. Separate logistic regressions were built for the independent variables capturing F0 estimates, fricative spectral moments, and formant estimates. Consistent with prior work on selective adoption (Table 1), we conceptualised F0, /s/, and vowel measures as domains that potentially index gay identity relatively independently; therefore, we tested F0, /s/, and vowel measures in separate models. In addition, these variables were extracted from different subsets of the speech samples. As a result, where one set of independent variables was estimated, the other two were non-applicable, preventing placing all acoustic measurements into one model.

The three logistic regression models were conceptualized as testing individual hypotheses regarding F0, /s/, and vowel production, as gay speakers can exhibit gender-nonconforming behavior in one aspect of their speech independently of other features (Munson et al., 2006; Pierrehumbert et al., 2004, see Table 1 for an overview). As each model must return at least one factor that significantly predicts sexual identity to reject the associated null hypothesis (i.e., there are no F0, fricative, or vowel differences between gay and straight speakers), alpha was not adjusted for multiple comparisons, despite the three regression models being built using the same speaker sample (Rubin, 2021).

In the F0 model, the independent variables were M, SD, and Range of F0 (numeric, non-interacting). In the fricative model, independent factors were Center of Gravity, Standard Deviation, Skewness, Kurtosis, and Duration (numeric, non-interacting). In the vowel formant model, independent factors were F1 and F2 (numeric) and Phoneme (categorical, with the levels /iː, e, oː ɐ, ɐː/, sum-coded comparing each level to the mean); interactions were included. Logistic regression models were built using *glm()* function with the *binomial* family in the *stats* package (R Core Team, 2021). For ease of interpreting effect size, estimates and standard errors were standardized using *z*-scoring with the *beta* function in the *reghelper* library and Cohen’s *d* was calculated using the *effectsize* library (Ben-Shachar et al., 2020; Hughes & Beiner, 2023).

Within-group differences for gay speakers were tested by linking survey variables to *z*-scored acoustic metrics using Pearson’s correlation in three separate correlation matrices for F0 estimates, fricative spectral moments, and formant estimates respectively. For each correlation matrix, *p*-values were calculated from Pearson’s *r* and adjusted for the number of acoustic measurement linked to each survey variable: when eight survey variables were linked to three F0 measures, alpha was adjusted as 0.05/3 = 0.016; when eight survey variables were linked to five /s/ measures, alpha was adjusted as 0.05/5 = 0.01; and when eight survey variables were linked to 12 vowel measures, alpha was adjusted as 0.05/12 = 0.004 (Rubin, 2021).

## Results

### Speaker Characteristics

The two speaker cohorts were compared along key survey variables (Table 4). Speakers identifying as gay on the categorical sexual identity question reported significantly higher scores for childhood gender-nonconformity, and lower scores for childhood and adulthood masculinity than speakers identifying as straight, consistent with the overall trend of higher gender-nonconformity among gay men (e.g., Allen & Robson, 2020; Bailey & Zucker, 1995).

**Table 4:** Demographic and psychological characteristics of the straight and gay speaker cohorts

Survey variable	Straight speakers M SD	Gay speakers M SD	*p* value	Cohen’s *d*
Age (years)	36 (15)	34 (12)	.449	0.19 (small)
Sexual Identity Continuum	1.0 (0)	4.8 (0.41)	<.001****	− 13.24 (large)
Childhood gender-nonconformity	2.0 (0.86)	3.6 (1.25)	<.001****	− 1.52 (large)
Childhood masculinity	5.8 (0.80)	4.6 (1.11)	<.001****	1.25 (large)
Adulthood masculinity	6.2 (0.69)	5.1 (1.25)	<.001****	1.07 (large)
Outness	Non-applicable	3.6 (1.13)	Non-applicable	Non-applicable
Straight-acting	Non-applicable	3.1 (1.15)	Non-applicable	Non-applicable
Internalized homophobia	Non-applicable	3.1 (1.08)	Non-applicable	Non-applicable

*p*-values show the results of paired *t*-tests with alpha adjusted to 0.01 for five comparisons using Bonferroni correction. Cohen’s *d* is presented to evaluate effect size interpreted as 0.20 is small effect, 0.50 is moderate, and 0.80 is large

### Acoustic Differences Between Straight and Gay Men

Results presented in this section address our first set of hypotheses that gay men would exhibit gender-nonconforming acoustic patterns relative to straight men (Munson et al., 2006). Results consistent with the hypotheses would show that gay men produce (1a) higher and more varied F0 with a larger range; (1b) /s/ with higher first, second, and fourth spectral moments, lower third spectral moment, and longer duration; and (1c) higher vowel formants.

#### F0 Characteristics as Predictors of Sexual Identity

**Fig. 2:**
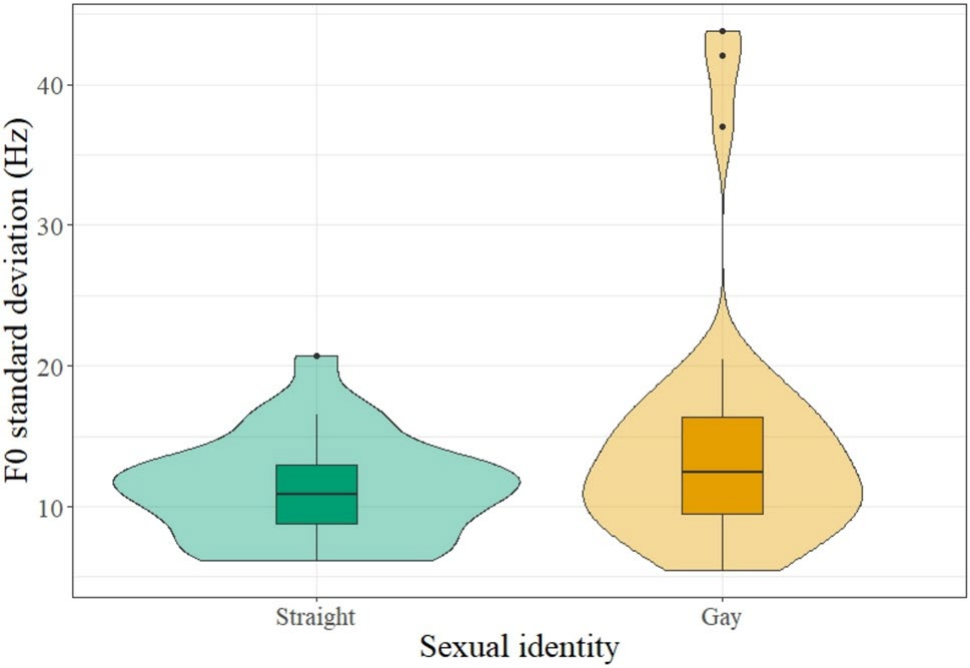
Standard deviation of F0 comparing straight (left, green) and gay (right, gold) male speakers of AusE

Consistent with Hypothesis (1a), standard deviation of F0 significantly predicted sexual identity (β = 1.756, SE = 0.82, z = 2.12, *p* = 0.034, Cohen’s *d* = 0.55; Fig. 2 and Table 5), such that greater F0 variability was associated with higher odds of gay identity. Mean F0 and F0 range did not reach statistical significance and showed estimates opposite the anticipated direction (mean F0: β = − 0.291, SE = 0.32, z = − 0.89, *p* = 0.373, Cohen’s *d* = − 0.23; F0 range: β = − 0.757, SE = 0.48, z = − 1.54, *p* = 0.121, Cohen’s *d* = − 0.40; Table 5).

**Table 5:** Results of logistic regressions capturing F0 differences between the straight and gay speaker cohorts

Independent variable	Estimate (non-standardized)	Estimate (standardized)	Standard Error	*z* value	*p* value	Cohen’s *d*
Intercept	1.40	0.51	0.31	1.63	0.1015	
Mean F0	− 0.01	− 0.29	0.32	− 0.89	0.373	− 0.23 (Small)
SD F0	0.23	1.75	0.82	2.12	0.0335	0.55 (Moderate)
Range F0	− 0.02	− 0.75	0.48	− 1.54	0.1213	− 0.40 (Small)
Null deviance	81.503 on 59 degrees of freedom					
Residual deviance	73.291 on 56 degrees of freedom					
χ^2^ with *p-value* calculated on 2 degrees of freedom	χ^2^ = 8.21 *p* = 0.0165*					

Estimates have been standardized by *z*-scoring numeric variables. Independent variables are evaluated for effect size using Cohen’s *d*, interpreted as 0.20 for small effect, 0.50 for moderate, and 0.80 for large. χ^2^ was calculated as the difference of null deviance and residual deviance (null – residual); *p*-value was calculated using χ^2^ and degrees of freedom of 2

Three gay speakers produced outliers with respect to their F0 standard deviation (F0 standard deviation exceeding the third quartile + 1.5 interquartile range), which may have driven the sexual orientation differences in F0 standard deviation reported above (Fig. 2). Therefore, data from the outliers were excluded and *p*-values were re-calculated. Excluding outliers did not change the significance of any of the predictors, but reduced effect size for F0 standard deviation (mean F0: *p* =0.381, Cohen’s *d* = − 0.23; F0 standard deviation: *p* =0.049, Cohen’s *d* = 0.51; range F0: *p* =0.131, Cohen’s *d* = − 0.39).

#### /s/ Characteristics as Predictors of Sexual Identity

**Fig. 3:**
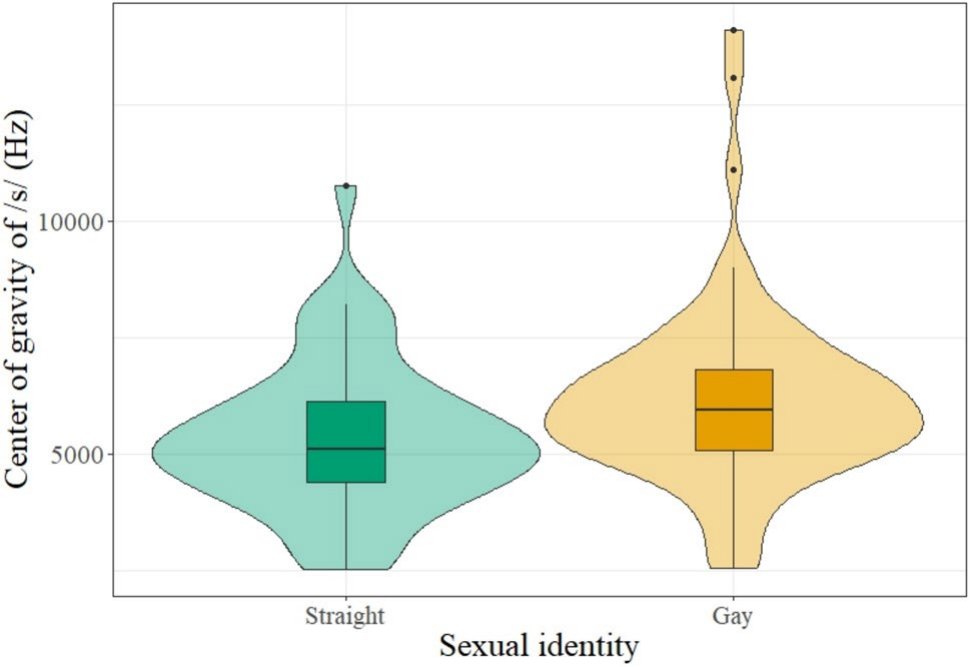
Center of gravity in /s/ comparing straight (left, green) and gay (right, gold) male speakers of AusE

Consistent with Hypothesis (1b), Center of Gravity significantly predicted sexual identity (β = 0.860, SE = 0.33, z = 2.58, *p* =0.009, Cohen’s *d* = 0.67; Fig. 3 and Table 6), such that higher center of gravity was associated with greater odds of gay identity. The remaining /s/ measures did not reach statistical significance and showed estimates opposite the anticipated direction (Standard Deviation: β = − 0.344, SE = 0.24, z = − 1.43, *p* =0.151, Cohen’s *d* = − 0.37; Skewness: β = 0.499, SE = 0.37, z = 1.35, *p* = 0.176, Cohen’s *d* = 0.37; Kurtosis: β = − 0.340, SE = 0.31, z = − 1.06, *p* = 0.286, Cohen’s *d* = − 0.28; Duration: β = − 0.031, SE = 0.20, z = − 0.15, *p* = 0.879, Cohen’s *d* = − 0.04; Table 6).

**Table 6:** Results of logistic regressions capturing fricative differences between the straight and gay speaker cohorts

Independent variable	Estimate (non-standardized)	Estimate (standardized)	Standard Error	z value	*p* value	Cohen’s *d*
Intercept	− 1.59	0.42	0.20	2.13	0.0326	
Center of Gravity	0.00	0.86	0.33	2.58	0.0098	0.67 (Moderate)
SD	− 0.00	− 0.34	0.23	− 1.43	0.1513	− 0.37 (Small)
Skewness	0.34	0.49	0.36	1.35	0.1764	0.35 (Small)
Kurtosis	− 0.02	− 0.33	0.31	−1.06	0.2861	− 0.28 (Small)
Duration	− 0.50	− 0.03	0.20	− 0.15	0.8797	− 0.04 (Negligible)
Null deviance	153.77 on 113 degrees of freedom					
Residual deviance	144.34 on 108 degrees of freedom					
χ^2^ with *p-value* calculated on 4 degrees of freedom	χ^2^ = 9.42 *p* = 0.513					

Estimates have been standardized by *z*-scoring numeric variables. Independent variables are evaluated for effect size using Cohen’s *d*, interpreted as 0.20 for small effect, 0.50 for moderate, and 0.80 for large. χ^2^ was calculated as the difference of null deviance and residual deviance (null—residual); *p*-value was calculated using χ^2^ and degrees of freedom of 5

Three gay speakers produced /s/ with center of Gravity outliers (Center of Gravity exceeding the third quartile + 1.5 interquartile range), which may have driven the sexual identity differences in /s/ Center of Gravity reported above (Fig. 3). Therefore, data from the outliers were excluded and *p*-values were re-calculated. Excluding outliers did not change the significance of any of the predictors or conventional effect sizes for Cohen’s *d* (Center of Gravity: *p* = 0.028, Cohen’s *d* = 0.57; Standard Deviation: *p* = 0.141, Cohen’s *d* = − 0.38; Skewness: *p* = 0.192, Cohen’s *d* = 0.34; Kurtosis: *p* = 0.273, Cohen’s *d* = − 0.28; Duration: *p* = 0.892, Cohen’s *d* = − 0.03).

#### Vowel Formants as Predictors of Sexual Identity

**Fig. 4:**
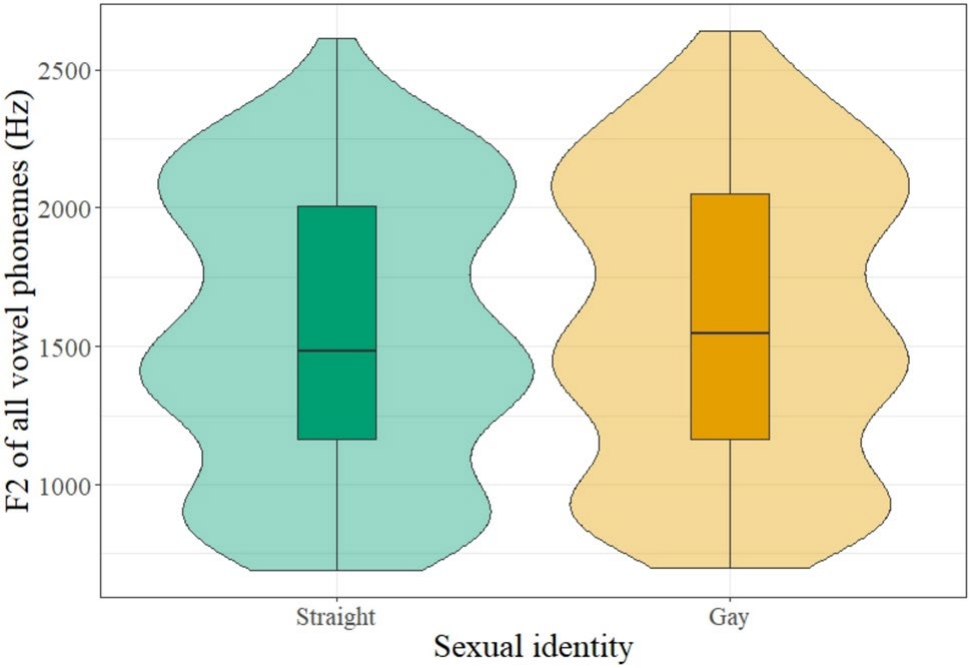
Overall F2 comparing straight (left, green) and gay (right, gold) male speakers of AusE

**Fig. 5:**
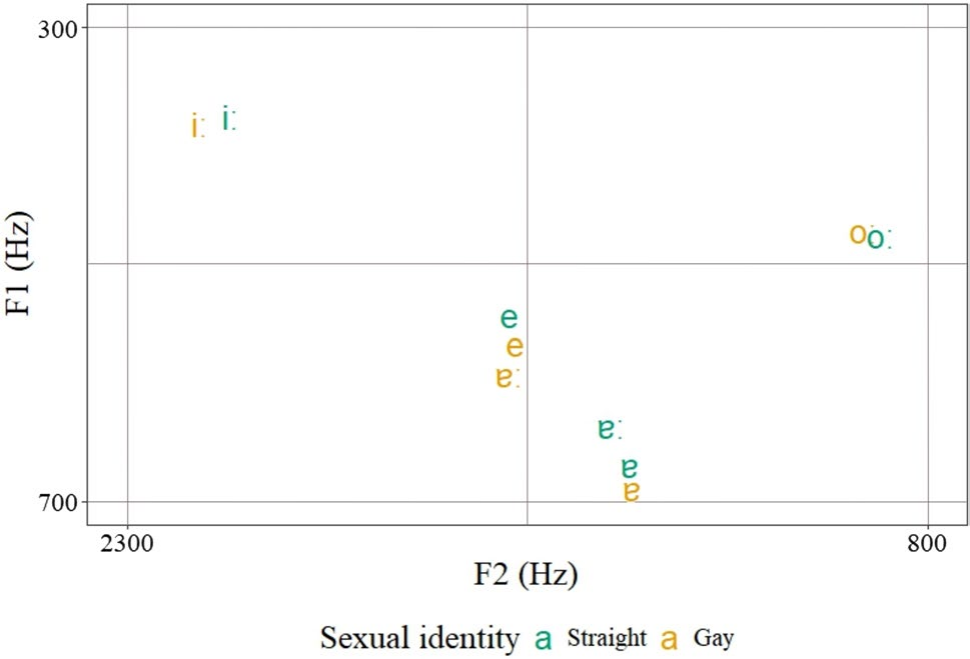
Vowel space of straight (green) and gay (gold) male speakers of AusE*. Note* F1 indicates vowel height (low value: high vowel). F2 indicates vowel frontness (high value: front vowel)

**Fig. 6:**
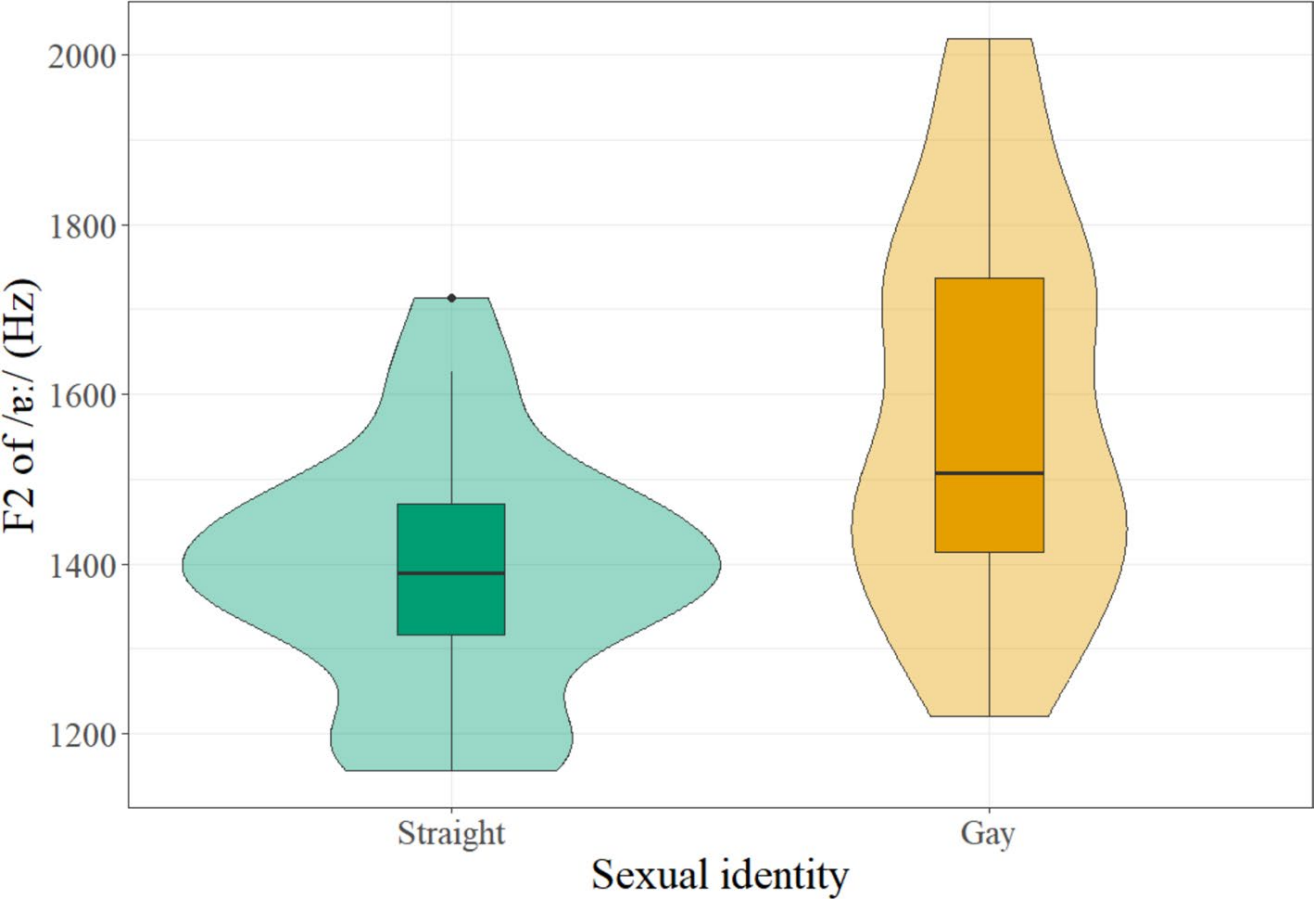
F2 of /ɐː/ comparing straight (left, green) and gay (right, gold) male speakers of AusE

Overall F1 did not reach statistical significance although the estimate was in the anticipated direction (β = 0.353, SE = 0.18, z = 1.91, *p* = 0.056, Cohen’s *d* = 0.49; Table 7). Overall F2 significantly predicted sexual identity (β = 0.637, SE = 0.25, z = 2.45, *p* = 0.014, Cohen’s *d* = 0.63; Figs. 4 and 5), indicating that higher F2 was associated with greater odds of gay identity. There was also evidence that the association between F2 and sexual identity varied by phoneme: the F2 × /ɐː/ interaction was significant (β = 1.428, SE = 0.54, z = 2.63, *p* = 0.008, Cohen’s *d* = 0.68; Fig. 6), suggesting a stronger positive association between F2 for /ɐː/ and gay identity relative to the mean effect across phonemes. The remaining F1 × Phoneme and F2 × Phoneme interactions did not reach statistical significance (*p* > 0.05; Table 7).

**Table 7:** Results of logistic regressions capturing fricative differences between the straight and gay speaker cohorts

Independent variable	Estimate (non-standardized)	Estimate (standardized)	Standard Error	*z* value	*p value*	Cohen’s *d*
Intercept	− 3.27	0.21	0.25	0.83	0.4032	
F1	0.00	0.35	0.18	1.91	0.0557	0.49 (Small)
F2	0.00	0.63	0.25	2.45	0.0141	0.63 (Moderate)
F1:Phoneme.iː	0.00	0.17	0.28	0.61	0.5365	0.16 (Negligible)
F1:Phoneme.e	0.00	0.10	0.24	0.44	0.6528	0.12 (Negligible)
F1:Phoneme.ɐ	0.00	− 0.10	0.16	− 0.61	0.5403	− 0.16 (Negligible)
F1:Phoneme.ɐː	− 0.00	0.15	0.19	0.81	0.4153	0.21 (Small)
F2:Phoneme.iː	− 0.00	− 0.07	0.36	− 0.21	0.8296	− 0.06 (Negligible)
F2:Phoneme.e	− 0.00	− 0.53	0.60	− 0.88	0.3781	− 0.23 (Small)
F2:Phoneme.ɐ	− 0.00	− 0.31	0.49	− 0.64	0.5166	− 0.17 (Negligible)
F2:Phoneme.ɐː	0.00	1.42	0.54	2.63	0.0084	0.68 (Moderate)
Null deviance	620.48 on 458 degrees of freedom					
Residual deviance	602.11 on 448 degrees of freedom					
χ^2^ with *p-value* calculated on 11 degrees of freedom	χ^2^ = 21.03 *p* = 0.0330*					

Estimates have been standardized by *z*-scoring numeric variables. Independent variables are evaluated for effect size using Cohen’s *d*, interpreted as 0.20 for small effect, 0.50 for moderate, and 0.80 for large. χ^2^ was calculated as the difference of null deviance and residual deviance (null—residual); *p*-value was calculated using χ^2^ and degrees of freedom of 10

### Acoustic Features, Gender-Nonconformity, and Other Aspects of Gay Identities

Results presented in this section address our second set of hypotheses that gay men would exhibit within-group variation such that (2a) exclusively gay identity; (2b) lower self-reported adulthood and childhood masculinity, higher childhood gender-nonconformity, and lower straight-acting identification; and (2c) higher outness and lower internalized homophobia would be linked to increased gender-nonconforming voice and speech behavior. Gender-nonconforming speech behavior were expected to be evidenced through higher and more varied F0 with a wider range, /s/ produced with higher first, second, and fourth spectral moments, lower third spectral moment, and longer duration, and vowels produced with higher formant values.

#### Correlations with F0

**Fig. 7:**
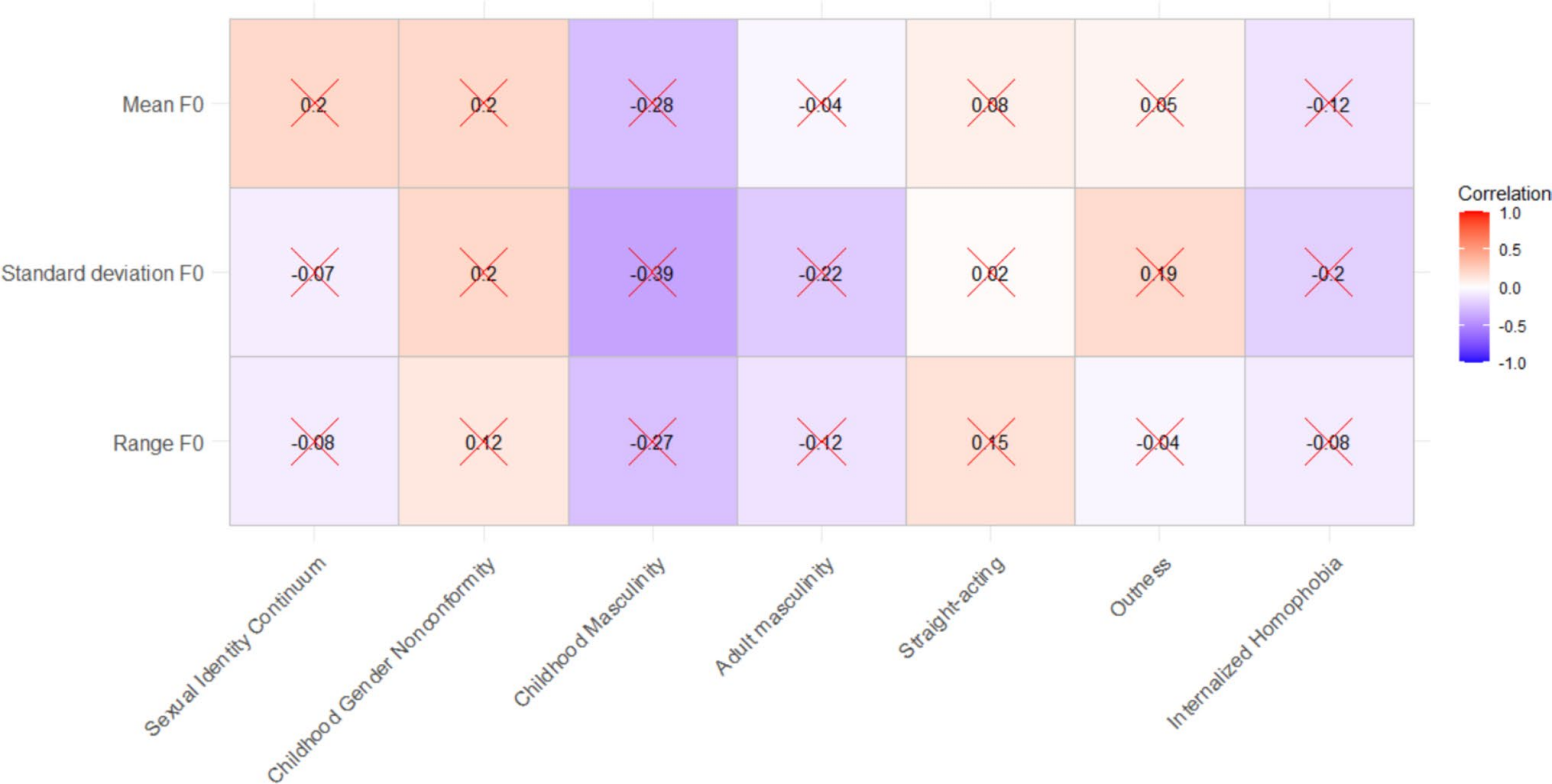
Correlations between gay speakers’ psychological characteristics (rows) and F0 estimates (columns). *Note*. Correlations are shown using Pearson’s *r*. Positive correlations are highlighted in red, negative correlations in purple. Darkness indicates strength of correlation. Non-significant correlations are crossed out; significance level was adjusted for 3 comparisons as 0.05/3. Note that all correlations are non-significant. (Color figure online)

None of the correlations between F0 metrics (mean, SD, range) and the psychological predictors reached the statistical significance threshold (Fig. 7). Correlation coefficients were not uniformly near zero, for example, lower childhood masculinity was non-significantly and moderately associated with more gender-nonconforming greater F0 variability (SD F0; *r* = −0.39; Fig. 7). The full set of correlations (including *r*, *n*, confidence intervals, and exact *p*-values with both unadjusted and corrected values) are reported in Supplementary Table S1.

#### Correlations with /s/ production

**Fig. 8:**
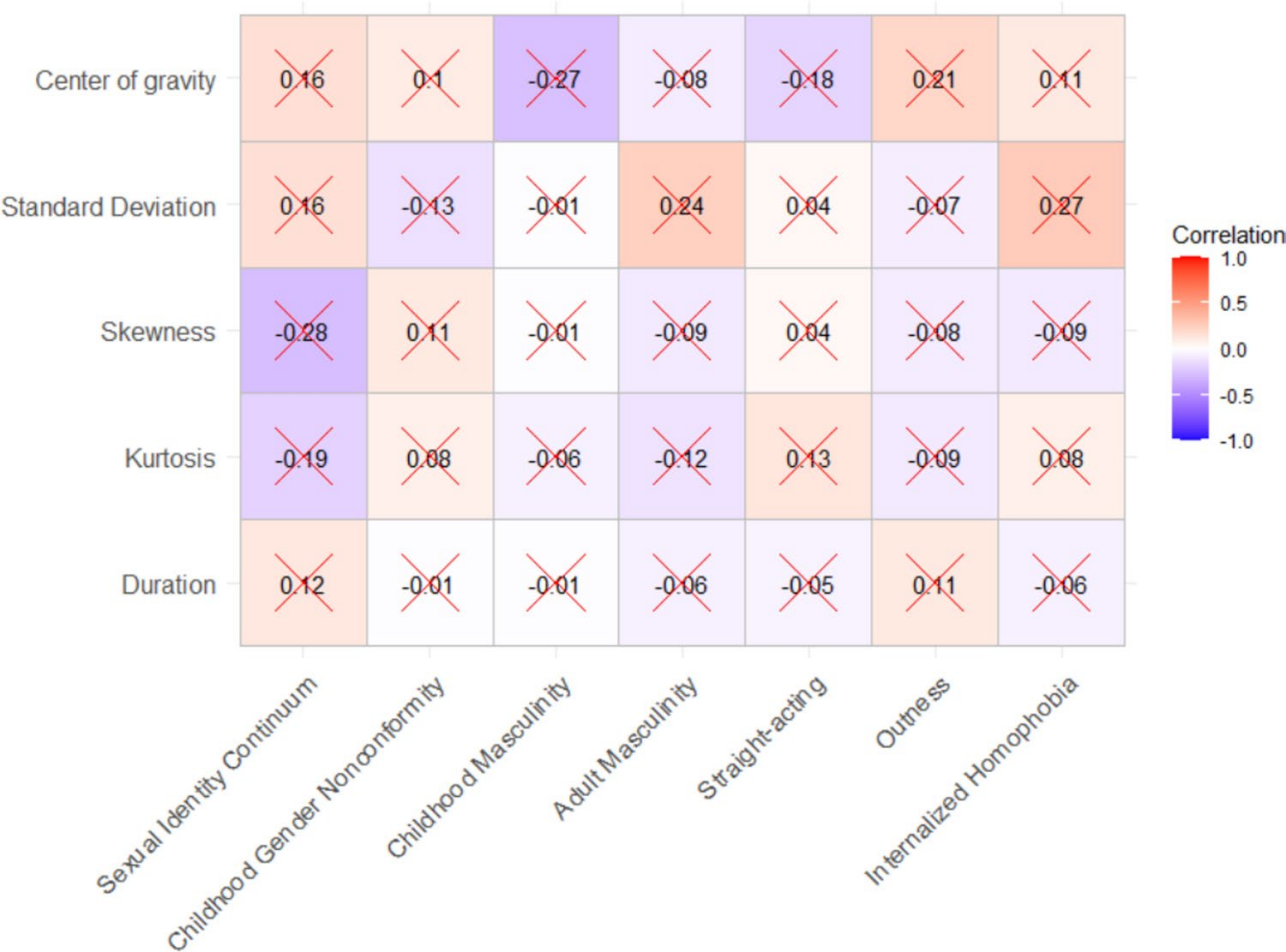
Correlations between gay speakers’ psychological characteristics (rows) and /s/ characteristics (columns). *Note* Correlations are shown using Pearson’s *r*. Positive correlations are highlighted in red, negative correlations in purple. Darkness indicates strength of correlation. Non-significant correlations are crossed out; significance level was adjusted for 5 comparisons as 0.05/5. Note that all correlations are non-significant. (Color figure online)

None of the correlations between /s/ measures and the psychological predictors reached the threshold of significance (Fig. 8). Correlation coefficients were not uniformly near zero; for example, exclusively gay identity showed a non-significant association with more gender-nonconforming, lower /s/ skewness (*r* = −0.28). Higher internalised homophobia, however, was non-significantly and positively associated with more gender-nonconforming, higher standard deviation in /s/ (*r* = 0.27). The full set of correlations (including *r*, *n*, confidence intervals, and exact *p*-values with both unadjusted and corrected values) are reported in Supplementary Table S2.

#### Correlations with F1 and F2

**Fig. 9:**
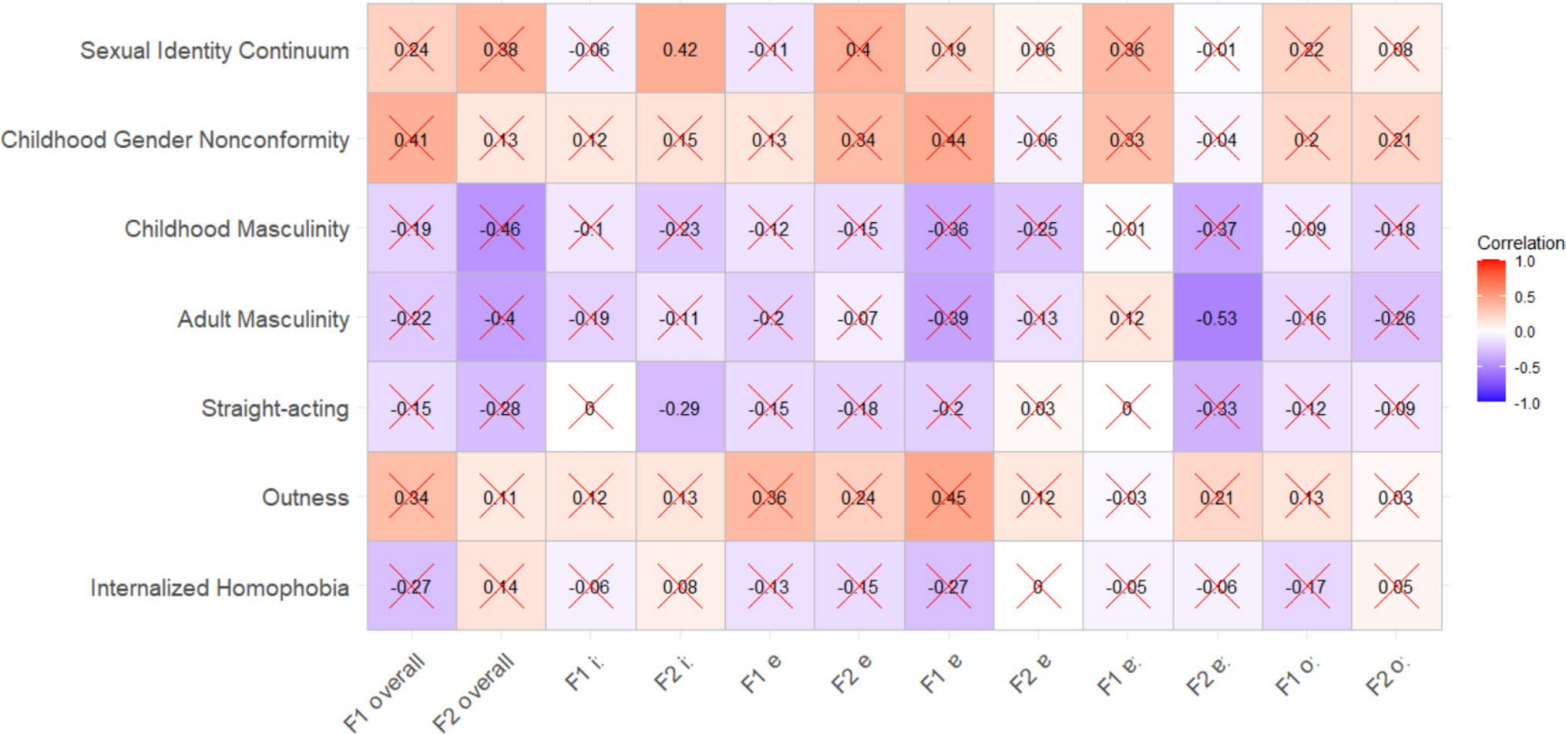
Correlations between gay speakers’ psychological characteristics (rows) and vowel formant estimates (columns). *Note* Correlations are shown using Pearson’s *r*. Positive correlations are highlighted in red, negative correlations in purple. Darkness indicates strength of correlation. Non-significant correlations are crossed out; significance level was adjusted for 12 comparisons as 0.05/12. (Color figure online)

Among gay speakers, some vowel formant measures were significantly associated with the psychological predictors (see Fig. 9). Consistent with the directional hypotheses, more exclusively gay sexual identity (higher scores on the continuous sexual identity measure) was associated with more gender-nonconforming higher F2 for /iː/ (Fig. 9).

Consistent with the hypothesised direction of masculinity, higher self-reported masculinity in adulthood as well as self-reported straight acting identification were significantly associated with more masculine vowel production (higher adult masculinity: lower F2 in /ɐː/, higher straight-acting identification: lower F2 in /iː/).

The remaining non-significant correlation coefficients were not uniformly near zero (Fig. 9). For example, increased childhood gender-nonconformity and lower childhood masculinity showed non-significant correlations with gender-nonconforming vowel production (childhood gender-nonconformity: higher F1 in /ɐ / (*r* = 0.44) and overall F1 (*r* = 0.41; lower childhood masculinity: higher overall F2 (*r* = −0.46)). The full set of correlations (including *r*, *n*, confidence intervals, and exact *p*-values with both unadjusted and corrected values) are reported in Supplementary Table S3.

## Discussion

This paper aimed to (1) examine speech differences between gay and straight speakers of AusE and (2) explore variation in the gay speech community based on their gender expression and attitudes to their sexual identity. We found that relative to straight speakers, gay speakers of AusE produced gender-nonconforming patterns via increased F0 variation; increased center of gravity in /s/; and overall increased F2, and further increased F2 in /ɐː/. We found no evidence of gender-nonconforming mean and range of F0, standard deviation, skewness, kurtosis, or duration of /s/, or in F1.

We found that gay speakers of AusE produced more gender-nonconforming vowels when they reported being exclusively gay as opposed to mostly gay; lower adulthood masculinity; and being less straight-acting. We did not find within-group variation among gay speakers in F0 patterns and /s/ production.

### Gender-Nonconforming Speech Patterns of Gay Men in AusE

When examining speech differences between gay and straight speakers of AusE, gay speakers were hypothesised to produce some gender-nonconforming speech patterns in their F0, /s/, and/or vowel production, without producing speech that is overall gender-nonconforming. Gay man shifted some of their speech features towards the feminine direction in AusE by producing four gender-nonconforming speech features relative to straight men: higher standard deviation of F0, higher center of gravity of /s/, and overall higher vowel F2, as well as in /ɐː/ (Cox, 2006; Leung et al., 2022; Stevens & Harrington, 2016). These results collectively are consistent with the selective adoption hypothesis proposing that gay men selectively exhibit some gender-nonconforming speech features without shifting all their speech features towards the feminine direction (Munson et al., 2006; Pierrehumbert et al., 2004).

Our results are broadly consistent with results on other accents of English, such that gay speakers may select the same gender-nonconforming features, leading to similarities between AusE gay speech and gay speech in other accents of English. For instance, more fronted /s/ production observed in the current study was also attested in American English (Linville, 1998; cf. with Munson et al., 2006). The non-significant effect in mean F0 in AusE parallels the lack of F0 differences between gay and straight speakers of American and Canadian English (Linville, 1998; Munson et al., 2006; Rendall et al., 2008).

#### F0 Characteristics of Gay Men’s Speech

We found mixed evidence regarding the relationship of F0 to sexual identity in men. Gay speakers produced F0 with greater standard deviation, a gender-nonconforming speech feature, compared to straight speakers, indicating larger variation in F0 over time. In contrast, gay men produced non-significantly more masculine (i.e., lower) F0. These results together suggest that overall F0 may not differ between gay and straight men, but rather straight men had their F0 tracks cluster around their mean more, while gay men tended to have their F0 tracks reach toward the lower extreme. F0 variation, however, was small for both sexual identities, potentially due to the short length of the speech sample. Collecting longer speech samples might have elicited larger F0 variation.

Auditory-visual observation of the acoustic signal suggested that varied F0 may have resulted from gay men producing creaky voice more often than straight men in the current sample. Creak can be identified visually as being different to clear tone and is known to reduce F0 relative to non-creaky voice (Keating et al., 2015; White et al., 2022). Thus, gay speakers alternating between creaky and non-creaky voice within the Australian anthem can explain increased variation in F0 by occasional lowering of F0. Yet, potentially increased creak production did not result in overall lower F0 for gay men. As gay men, in particular younger gay men and gay men who agree with traditional gender roles, produce more creak than straight men (Shea et al., 2024), future research is required to examine the links between F0 variation, creaky voice, gay identity, and masculinity.

Differences in mean and range of F0 of gay and straight were such that gay men produced lower F0 and narrower range, although these differences were statistically non-significant. The non-significant result adds to the growing body of studies failing to find significant difference in overall F0 in varieties of English and other languages, such as Italian, German, and Czech (Kachel et al., 2018; Linville, 1998; Munson et al., 2006; Rendall et al., 2008; Sulpizio et al., 2015; Valentova & Havlíček, 2013). Despite the lack of evidence for significantly gender-nonconforming F0 production, listeners consistently perceive male speakers producing higher F0 as gay and more feminine, indicating a potential stereotype inaccuracy on the listeners’ behalf (Gaudio, 1994; Levon, 2007; Munson, 2007; Valentova & Havlíček, 2013).

#### /s/ Characteristics of Gay Men’s Speech

The present study found that relative to straight men, gay men produced more gender-nonconforming /s/ (i.e., with higher frequencies) in AusE. This is consistent with previous findings in American English and Italian (Linville, 1998; cf. with Munson et al., 2006; Sulpizio et al., 2015). While gay speakers’ /s/ production is skewed towards female-typical speech, the magnitude of difference between gay and straight speakers is relatively small with 800 Hz, whereas AusE-speaking women produce /s/ with approximately 3500 Hz higher center of gravity than AusE-speaking men (Stevens & Harrington, 2016).

#### Vowel Characteristics of Gay Men’s Speech

Relative to straight men, gay speakers produced vowels with significantly higher average F2. In addition, F2 was further increased in /ɐː/, which was also produced with a noticeably but non-significantly lower F1 by gay speakers (Fig. 5). That is, /ɐː /was characterised by a significantly higher F2, which is considered gender-nonconforming, and non-significantly lower F1, which is considered gender-conforming (Cox, 2006; Munson & Babel, 2019; Munson et al., 2006). The apparent contradiction can be resolved by considering the phonetic environment in which the formants of /ɐː/ were estimated. The vowel /ɐː/ is canonically produced with high first and low second formant (Cox & Fletcher, 2017). In the current study, /ɐː/ formants were estimated in the phrase *we are young* (/wiː ɐː jɐŋ/)^3^, in which /ɐː/ is preceded by /iː/ and followed by /j/. The phonemes /iː/ and /j/ are canonically produced with low F1 and high F2 values. When /ɐː/ is coarticulated with /iː/ and /j/, it becomes acoustically similar to the adjacent phonemes, leading to a reduction in first formant and an increase in the second formant of /ɐː/.

That is, gay speakers producing /ɐː/ with potentially lower F1 and higher F2 might be consistent with gay speakers coarticulating the vowel more with its phonetic context than straight speakers. More coarticulation in AusE would contrast with the observation that gay speakers produce clear speech in American English, highlighting accent differences in gay speech (Munson et al., 2006). As the current data are limited to a single token of /ɐː/, more research is required to clarify these findings.

### Speech Variation Within the Gay Community: Gender-Nonconformity and Other Aspects of Identity

We examined if gender-nonconforming voice and speech production is linked to gay speakers’ psychological profile. We did not find evidence for vocal fundamental frequency and /s/ -production varying with the sexual identity continuum, gender expression, outness, nor internalised homophobia among gay speakers, providing no evidence for our second set of hypotheses.

Vowel production was found to be a marker of gay identity and masculinity among gay speakers of AusE. The vowel /iː/ (*we, free, sea*) was produced with more gender-nonconforming (i.e., higher) F2 by speakers who identified as exclusively gay over mostly gay and who identified as less-straight acting, and thus identified less with traditional, heteronormative masculinity (Hunt et al., 2020). The vowel /ɐː/ (*are*) was produced with more gender-nonconforming (i.e., higher) F2 by speakers who identified as less masculine and also indexed gay-straight differences. As both vowels simultaneously index gay sexual identity and gender-nonconformity, it is possible that these gender-nonconforming vowels produced by gay men are linked to and an expression of overall increased gender-nonconforming behavior among gay men.

Similarly, the non-significant moderate association between lower childhood masculinity and increased F0 variability (Fig. 7) is consistent with the increased F0 variation in gay men’s speech being modulated by masculinity, given that gay men reported being less masculine than the straight men in the current sample. A potential link between lower childhood masculinity and adult gender-nonconforming speech behavior may also warrant future research on whether gender-nonconforming children selectively attend and adopt certain speech features of the opposite sex during early language acquisition and whether gender-nonconforming speech features emerge in childhood, similarly to gender-conforming features (Pierrehumbert et al., 2004; Rieger et al., 2008; Wong et al., 2025).

The effects of sexual identity and gender-nonconformity on speech were examined separately. Gay speakers in our sample reported higher scores for childhood gender-nonconformity, and lower scores for childhood and adulthood masculinity relative to the straight speakers, consistent with the overall trend of higher gender-nonconformity among gay men (e.g., Allen & Robson, 2020; Bailey & Zucker, 1995). Therefore, it is possible that differences in F0 and /s/ production between gay and straight men are at least partially driven by gay men being more gender-nonconforming (e.g., Allen & Robson, 2020; Bailey & Zucker, 1995). This is further supported by variation in vowel production among gay men simultaneously indexing sexual identity and masculinity. Future interdisciplinary research is required to address the complex interactions between sexual identity, gender expression, and voice and speech production in Australian English.

### Limitations

The main limitation of the current study was the method of speech sample collection. While on-line data collection with audio-samples recorded using participants’ own smartphones had the advantage of reaching a large number of potential participants and led to successful questionnaire data collection from 2133 participants, only 472 (22%) uploaded a voice sample, out of which only 60 (2.8%) passed quality screening. While care was taken to only include high quality recordings, different recording devices are likely to have introduced uncontrolled variation to the data (De Decker & Nycz, 2011; Leemann et al., 2020; Penney et al., 2021; Rathcke et al., 2017). For example, vowel formants vary between recording devices, indicating that ideally, device specifications should be the same for all participants (De Decker & Nycz, 2011; Guan & Li, 2021; Penney et al., 2021; Rathcke et al., 2017; Zhang et al., 2021). Despite these challenges, smartphones have provided data suitable for acoustic vowel analysis as early as 2011 (De Decker & Nycz, 2011) with F1 and F2 values measured in lossy compressions falling within 3–9 Hz of values measured in uncompressed data (Bulgin et al., 2010). F0 measures were not found to differ between recording devices (Guan & Li, 2021; Rathcke et al., 2017; Zhang et al., 2021).

On-line data collection also placed limitations on the stimulus. The Australian anthem was chosen as text that participants were expected to be able to recite without having to read from the screen. This led to a short and phonetically unbalanced stimulus. A longer text (e.g., the *Arthur, the Rat* passage) would have allowed for capturing range and variation in F0 more accurately (Leung et al., 2022). A phonetically balanced stimulus would have allowed for having a balanced sample with at least three repetitions of key vowel phonemes and /s/, in canonical contexts that control for coarticulation. Future research should aim to further examine variation in the speech of gay AusE-speaking men in lab-based recordings with a phonetically balanced stimulus.

Given the phonetically unbalanced stimulus and the large number of factors examined, smaller but potentially meaningful effects may not have reached the threshold of statistical significance. Therefore, effect size was reported for all results to provide targets for future preregistered studies designed with sufficient power to detect associations of approximately |*r*| ≈0.30–0.40 under multiple-testing control.

### Conclusion

We examined acoustic differences between gay and straight speakers of Australian English, a variety of English with only two perceptual and two acoustic studies on gay speech (Morandini et al., 2023; Shea et al., 2023, 2024; Szalay et al., 2022). The key markers of gay identity in AusE were vocal fundamental frequency produced with larger variation and /s/ produced with higher center of gravity. Vowel production showed complex differences between gay and straight speakers: while gay speakers produced vowels with gender-nonconforming second formants, results were complicated by vowel-consonant coarticulation. As the gay speakers were overall more gender-nonconforming than the straight speakers, it is possible, that gender-nonconforming speech features were linked to overall gender-nonconformity.

We also examined if gay men’s speech shows acoustic variation corresponding to their gender expression and attitudes to their own sexual identity. Gender-nonconforming vowels were produced by gay speakers reporting being less masculine and less straight-acting. That is, gender-nonconforming speech features in AusE-speaking gay men indexed sexual identity as well as gender-nonconformity. This result is overall consistent with gay men showing more gender-nonconforming behaviors compared to straight men (Allen & Robson, 2020; Bailey & Zucker, 1995), as well as with the cultural diversity observed in gay speech (Holmes et al., 2024; Munson et al., 2006; Pierrehumbert et al., 2004; Rendall et al., 2008).

## Supplementary Information

Below is the link to the electronic supplementary material.

Supplementary file1 (PDF 206 kb)

## Data Availability

Audio data are not available to protect participants’ privacy. Anonymised acoustic features and survey data are available from the corresponding author upon request.

## Footnotes

1 As per the guidelines of the American Psychological Association, we avoided the outdated term “homosexual” and chose the word “gay” to refer to men who identify as exclusively or predominantly gay (Kinsey et al., 1948; Veldhuis et al., 2024). To achieve stylistic consistency with “gay,” the word “straight” was chosen over “heterosexual” to describe individuals who identify as exclusively or predominantly straight (Kinsey et al., 1948). Men identifying as bisexual, pansexual, queer, or with other sexual orientations are not included in our terminology of “gay.”

2 The symbols used are those proposed by Harrington et al. (1997) for AusE.

3 Note that AusE is a non-rhotic accent; thus, no/ɹ/is produced.

## Code Availability

Available from the corresponding author upon request.
