# Supplementary material for: Sexual Identity, Gender-Nonconformity, and Acoustic Speech Characteristics of Gay and Straight Australian English-Speaking Men
Source: Arch Sex Behav. 2026 Jun 27;55(5):1921–39. doi: 10.1007/s10508-026-03468-4 (PMC13427862; doi:10.1007/s10508-026-03468-4)
Supplement: Supplementary file 1 — Supplementary file1 (PDF 206 kb) [file 10508_2026_3468_MOESM1_ESM.pdf]

Table S1

Correlations between psychological characteristics and F0 metrics among gay speakers (Figure 7).

Predictor	Acoustic measure	R	N	95% CI LL	95% CI UL	*p* (raw)	*p* (Adjusted)
Sexual identity continuum	Mean F0	0.20	35	-0.15	0.50	0.258	0.773
Childhood gender nonconformity	Mean F0	0.20	35	-0.14	0.50	0.243	0.729
Childhood masculinity	Mean F0	-0.28	35	-0.56	0.06	0.109	0.328
Adult masculinity	Mean F0	-0.04	35	-0.37	0.30	0.822	1.000
Straight-acting	Mean F0	0.08	35	-0.26	0.40	0.655	1.000
Outness	Mean F0	0.05	35	-0.29	0.38	0.771	1.000
Internalized homophobia	Mean F0	-0.12	35	-0.43	0.22	0.501	1.000
Sexual identity continuum	SD F0	-0.07	35	-0.39	0.27	0.707	1.000
Childhood gender nonconformity	SD F0	0.20	35	-0.15	0.50	0.258	0.776
Childhood masculinity	SD F0	-0.39	35	-0.64	-0.06	0.021	0.064
Adult masculinity	SD F0	-0.22	35	-0.52	0.12	0.199	0.598
Straight-acting	SD F0	0.02	35	-0.31	0.35	0.900	1.000
Outness	SD F0	0.19	35	-0.15	0.50	0.263	0.790
Internalized homophobia	SD F0	-0.20	35	-0.50	0.14	0.247	0.742
Sexual identity continuum	Range F0	-0.08	35	-0.40	0.26	0.650	1.000
Childhood gender nonconformity	Range F0	0.12	35	-0.23	0.43	0.507	1.000
Childhood masculinity	Range F0	-0.27	35	-0.55	0.07	0.121	0.363
Adult masculinity	Range F0	-0.12	35	-0.43	0.23	0.506	1.000
Straight-acting	Range F0	0.15	35	-0.20	0.46	0.397	1.000
Outness	Range F0	-0.04	35	-0.37	0.30	0.833	1.000
Internalized homophobia	Range F0	-0.08	35	-0.40	0.26	0.655	1.000

Note. Pearson correlations (r) were transcribed from the heatmap figures. N = 35 for all correlations. 95% confidence intervals were computed using Fisher's z transformation. Two-tailed p values were computed from r and n. Adjusted *p*-values were computed by multiplying the raw *p* by m (Figure 7: m = 3 F0 outcomes), capped at 1.00.

Table S2

Correlations between psychological characteristics and /s/ acoustic among gay speakers (Figure 8).

Predictor	Acoustic measure	R	N	95% CI LL	95% CI UL	*p* (raw)	*p* (Adjusted)
Sexual identity continuum	CoG	0.16	68	-0.08	0.39	0.183	0.916
Childhood gender nonconformity	CoG	0.10	68	-0.15	0.33	0.434	1.00
Childhood masculinity	CoG	-0.27	68	-0.48	-0.03	0.027	0.137
Adult masculinity	CoG	-0.08	68	-0.31	0.16	0.508	1.00
Straight-acting	CoG	-0.18	68	-0.4	0.06	0.141	0.703
Outness	CoG	0.21	68	-0.03	0.43	0.08	0.4
Internalized homophobia	CoG	0.11	68	-0.13	0.34	0.362	1.00
Sexual identity continuum	SD	0.16	68	-0.08	0.38	0.2	0.999
Childhood gender nonconformity	SD	-0.13	68	-0.35	0.12	0.305	1.00
Childhood masculinity	SD	-0.01	68	-0.25	0.23	0.937	1.00
Adult masculinity	SD	0.24	68	0	0.45	0.049	0.243
Straight-acting	SD	0.04	68	-0.2	0.28	0.733	1.00
Outness	SD	-0.07	68	-0.31	0.17	0.55	1.00
Internalized homophobia	SD	0.27	68	0.03	0.48	0.026	0.13
Sexual identity continuum	Skewness	-0.28	68	-0.49	-0.04	0.021	0.104
Childhood gender nonconformity	Skewness	0.11	68	-0.13	0.34	0.353	1.00
Childhood masculinity	Skewness	-0.01	68	-0.25	0.23	0.942	1.00
Adult masculinity	Skewness	-0.09	68	-0.32	0.15	0.457	1.00
Straight-acting	Skewness	0.04	68	-0.2	0.27	0.755	1.00
Outness	Skewness	-0.08	68	-0.31	0.16	0.535	1.00
Internalized homophobia	Skewness	-0.09	68	-0.32	0.16	0.484	1.00
Sexual identity continuum	Kurtosis	-0.19	68	-0.41	0.05	0.125	0.625
Childhood gender nonconformity	Kurtosis	0.08	68	-0.16	0.31	0.504	1.00
Childhood masculinity	Kurtosis	-0.06	68	-0.29	0.18	0.641	1.00
Adult masculinity	Kurtosis	-0.12	68	-0.35	0.12	0.324	1.00
Straight-acting	Kurtosis	0.13	68	-0.11	0.36	0.287	1.00

Outness	Kurtosis	-0.09	68	-0.32	0.15	0.481	1.00
Internalized homophobia	Kurtosis	0.08	68	-0.16	0.32	0.498	1.00
Sexual identity continuum	Duration	0.12	68	-0.13	0.34	0.35	1.00
Childhood gender nonconformity	Duration	-0.01	68	-0.25	0.23	0.949	1.00
Childhood masculinity	Duration	-0.01	68	-0.24	0.23	0.956	1.00
Adult masculinity	Duration	-0.06	68	-0.3	0.18	0.601	1.00
Straight-acting	Duration	-0.05	68	-0.29	0.19	0.664	1.00
Outness	Duration	0.11	68	-0.14	0.34	0.387	1.00
Internalized homophobia	Duration	-0.06	68	-0.29	0.18	0.624	1.00

Note. Pearson correlations (r) were transcribed from the heatmap figures. N = 68 for all correlations. 95% confidence intervals were computed using Fisher's z transformation. Two-tailed p values were computed from r and n. Adjusted *p*-values were computed by multiplying the raw *p* by m (Figure 8: m = 5 /s/ outcomes), capped at 1.00.

Table S3

Correlations between psychological characteristics and vowel formant estimates among gay speakers (Figure 9).

**Predictor**	**Acoustic measure**	**R**	**N**	**95% CI LL**	**95% CI UL**	***p* (raw)**	***p* (Adjusted)**
Sexual identity continuum	F1 overall	0.24	35	-0.1	0.53	0.162	1.00
Childhood gender nonconformity	F1 overall	0.41	35	0.09	0.65	0.014	0.171
Childhood masculinity	F1 overall	-0.19	35	-0.49	0.16	0.286	1.00
Adult masculinity	F1 overall	-0.22	35	-0.51	0.13	0.211	1.00
Straight-acting	F1 overall	-0.15	35	-0.46	0.19	0.378	1.00
Outness	F1 overall	0.34	35	0.01	0.6	0.047	0.567
Internalized homophobia	F1 overall	-0.27	35	-0.55	0.07	0.12	1.00
Sexual identity continuum	F2 overall	0.38	35	0.05	0.63	0.026	0.314
Childhood gender nonconformity	F2 overall	0.13	35	-0.22	0.44	0.467	1.00
Childhood masculinity	F2 overall	-0.46	35	-0.69	-0.15	0.006	0.066
Adult masculinity	F2 overall	-0.4	35	-0.65	-0.08	0.017	0.202
Straight-acting	F2 overall	-0.28	35	-0.56	0.06	0.108	1.00
Outness	F2 overall	0.11	35	-0.23	0.43	0.54	1.00
Internalized homophobia	F2 overall	0.14	35	-0.2	0.45	0.426	1.00
Sexual identity continuum	F1 /i:/	-0.06	101	-0.25	0.13	0.536	1.00
Childhood gender nonconformity	F1 /i:/	0.12	101	-0.08	0.31	0.235	1.00
Childhood masculinity	F1 /i:/	-0.1	101	-0.29	0.1	0.337	1.00
Adult masculinity	F1 /i:/	-0.19	101	-0.37	0.01	0.062	0.741
Straight-acting	F1 /i:/	0	101	-0.2	0.2	0.999	1.00
Outness	F1 /i:/	0.12	101	-0.08	0.31	0.233	1.00
Internalized homophobia	F1 /i:/	-0.06	101	-0.25	0.14	0.557	1.00
Sexual identity continuum	F2 /i:	0.42	101	0.24	0.57	<0.001	0.00012
Childhood gender nonconformity	F2 /i:	0.15	101	-0.05	0.33	0.142	1.00
Childhood masculinity	F2 /i:	-0.23	101	-0.4	-0.03	0.023	0.274
Adult masculinity	F2 /i:	-0.11	101	-0.3	0.09	0.281	1.00
Straight-acting	F2 /i:	-0.29	101	-0.46	-0.1	0.004	0.045
Outness	F2 /i:	0.13	101	-0.06	0.32	0.186	1.00
Internalized homophobia	F2 /i:	0.08	101	-0.11	0.27	0.408	1.00

Sexual identity continuum	F1 /e/	-0.11	33	-0.44	0.24	0.533	1.00
Childhood gender nonconformity	F1 /e/	0.13	33	-0.23	0.45	0.488	1.00
Childhood masculinity	F1 /e/	-0.12	33	-0.44	0.24	0.523	1.00
Adult masculinity	F1 /e/	-0.2	33	-0.5	0.16	0.276	1.00
Straight-acting	F1 /e/	-0.15	33	-0.47	0.2	0.395	1.00
Outness	F1 /e/	0.36	33	0.02	0.63	0.037	0.45
Internalized homophobia	F1 /e/	-0.13	33	-0.45	0.23	0.486	1.00
Sexual identity continuum	F2 /e/	0.4	33	0.07	0.66	0.02	0.244
Childhood gender nonconformity	F2 /e/	0.34	33	0	0.61	0.05	0.6
Childhood masculinity	F2 /e/	-0.15	33	-0.47	0.21	0.414	1.00
Adult masculinity	F2 /e/	-0.07	33	-0.4	0.28	0.714	1.00
Straight-acting	F2 /e/	-0.18	33	-0.49	0.17	0.317	1.00
Outness	F2 /e/	0.24	33	-0.11	0.54	0.181	1.00
Internalized homophobia	F2 /e/	-0.15	33	-0.47	0.21	0.412	1.00
Sexual identity continuum	F1 /ɐ/	0.19	35	-0.16	0.49	0.285	1.00
Childhood gender nonconformity	F1 /ɐ/	0.44	35	0.13	0.68	0.008	0.091
Childhood masculinity	F1 /ɐ/	-0.36	35	-0.62	-0.03	0.033	0.392
Adult masculinity	F1 /ɐ/	-0.39	35	-0.64	-0.07	0.019	0.231
Straight-acting	F1 /ɐ/	-0.2	35	-0.5	0.14	0.243	1.00
Outness	F1 /ɐ/	0.45	35	0.13	0.68	0.007	0.085
Internalized homophobia	F1 /ɐ/	-0.27	35	-0.56	0.07	0.113	1.00
Sexual identity continuum	F2 /ɐ/	0.06	35	-0.28	0.39	0.722	1.00
Childhood gender nonconformity	F2 /ɐ/	-0.06	35	-0.39	0.28	0.732	1.00
Childhood masculinity	F2 /ɐ/	-0.25	35	-0.54	0.09	0.15	1.00
Adult masculinity	F2 /ɐ/	-0.13	35	-0.45	0.21	0.44	1.00
Straight-acting	F2 /ɐ/	0.03	35	-0.31	0.36	0.881	1.00
Outness	F2 /ɐ/	0.12	35	-0.22	0.44	0.489	1.00
Internalized homophobia	F2 /ɐ/	0	35	-0.33	0.33	0.996	1.00
Sexual identity continuum	F1 /ɐː/	0.36	35	0.03	0.62	0.033	0.394
Childhood gender nonconformity	F1 /ɐː/	0.33	35	0	0.6	0.051	0.607
Childhood masculinity	F1 /ɐː/	-0.01	35	-0.34	0.32	0.944	1.00
Adult masculinity	F1 /ɐː/	0.12	35	-0.22	0.44	0.492	1.00
Straight-acting	F1 /ɐː/	0	35	-0.33	0.33	0.995	1.00

Outness	F1 /ɐː/	-0.03	35	-0.36	0.31	0.872	1.00
Internalized homophobia	F1 /ɐː/	-0.05	35	-0.38	0.28	0.756	1.00
Sexual identity continuum	F2 /ɐː/	-0.01	35	-0.34	0.33	0.975	1.00
Childhood gender nonconformity	F2 /ɐː/	-0.04	35	-0.37	0.3	0.812	1.00
Childhood masculinity	F2 /ɐː/	-0.37	35	-0.62	-0.04	0.03	0.366
Adult masculinity	F2 /ɐː/	-0.53	35	-0.73	-0.24	0.001	0.013
Straight-acting	F2 /ɐː/	-0.33	35	-0.59	0.01	0.056	0.677
Outness	F2 /ɐː/	0.21	35	-0.13	0.51	0.226	1.00
Internalized homophobia	F2 /ɐː/	-0.06	35	-0.39	0.28	0.716	1.00
Sexual identity continuum	F1 /oː/	0.22	68	-0.02	0.44	0.068	0.812
Childhood gender nonconformity	F1 /oː/	0.2	68	-0.04	0.42	0.102	1.00
Childhood masculinity	F1 /oː/	-0.09	68	-0.32	0.15	0.453	1.00
Adult masculinity	F1 /oː/	-0.16	68	-0.39	0.08	0.18	1.00
Straight-acting	F1 /oː/	-0.12	68	-0.35	0.12	0.336	1.00
Outness	F1 /oː/	0.13	68	-0.12	0.35	0.306	1.00
Internalized homophobia	F1 /oː/	-0.17	68	-0.39	0.07	0.16	1.00
Sexual identity continuum	F2 /oː/	0.08	68	-0.16	0.32	0.499	1.00
Childhood gender nonconformity	F2 /oː/	0.21	68	-0.03	0.43	0.08	0.961
Childhood masculinity	F2 /oː/	-0.18	68	-0.4	0.06	0.145	1.00
Adult masculinity	F2 /oː/	-0.26	68	-0.47	-0.03	0.029	0.353
Straight-acting	F2 /oː/	-0.09	68	-0.33	0.15	0.443	1.00
Outness	F2 /oː/	0.03	68	-0.21	0.27	0.778	1.00
Internalized homophobia	F2 /oː/	0.05	68	-0.19	0.29	0.658	1.00

Note. Pearson correlations (r) were transcribed from the heatmap figures. N varies according to vowel phoneme. 95% confidence intervals were computed using Fisher's z transformation. Two-tailed p values were computed from r and n. Adjusted *p*-values were computed by multiplying the raw *p* by m (Figure 9: m = 12 vowel outcomes), capped at 1.00.
